# Evolutionary trajectories of tooth histology patterns in modern sharks (Chondrichthyes, Elasmobranchii)

**DOI:** 10.1111/joa.13145

**Published:** 2019-12-22

**Authors:** Patrick L. Jambura, Julia Türtscher, René Kindlimann, Brian Metscher, Cathrin Pfaff, Sebastian Stumpf, Gerhard W. Weber, Jürgen Kriwet

**Affiliations:** ^1^ Department of Palaeontology University of Vienna Vienna Austria; ^2^ Haimuseum und Sammlung R. Kindlimann Aathal‐Seegräben Switzerland; ^3^ Department of Theoretical Biology University of Vienna Vienna Austria; ^4^ Department of Evolutionary Anthropology University of Vienna Vienna Austria; ^5^ Core Facility for Micro‐Computed Tomography University of Vienna Vienna Austria

**Keywords:** dentition, histotype, micro‐computed tomography, teeth, tooth histology

## Abstract

During their evolutionary history, modern sharks developed different tooth mineralization patterns that resulted in very distinct histological patterns of the tooth crown (histotypes). To date, three different tooth histotypes have been distinguished: (i) orthodont teeth, which have a central hollow pulp cavity in the crown, encapsulated by a prominent layer of dentine (orthodentine); (ii) pseudoosteodont teeth, which have their pulp cavities secondarily replaced by a dentinal core of porous dentine (osteodentine), encased by orthodentine; and (iii) osteodont teeth, which lack orthodentine and the whole tooth crown of which consists of osteodentine. The aim of the present study was to trace evolutionary trends of tooth mineralization patterns in modern sharks and to find evidence for the presence of phylogenetic or functional signals. High resolution micro‐computed tomography images were generated for the teeth of members of all nine extant shark orders and the putative stem group †Synechodontiformes, represented here by three taxa, to examine the tooth histology non‐destructively. Pseudoosteodonty is the predominant state among modern sharks and represents unambiguously the plesiomorphic condition. Orthodonty evolved several times independently in modern sharks, while the osteodont tooth histotype is only developed in lamniform sharks. The two shark orders Heterodontiformes and Pristiophoriformes showed highly modified tooth histologies, with *Pristiophorus* exhibiting a histology only known from batomorphs (i.e. rays and skates), and *Heterodontus* showing a histological difference between anterior and posterior teeth, indicating a link between its tooth morphology, histology and durophagous lifestyle. The tooth histotype concept has proven to be a useful tool to reflect links between histology, function and its taxonomic value for distinct taxa; however, a high degree of variation, especially in the pseudoosteodont tooth histotype, demonstrates that the current histotype concept is too simplistic to fully resolve these relationships. The vascularization pattern of the dentine might offer new future research pathways for better understanding functional and phylogenetic signals in the tooth histology of modern sharks.

## Graphical abstract

Using micro‐computed tomography techniques, we examined the tooth mineralization in modern shark clades (Elasmobranchii, Chondrichthyes). The inclusion of fossil taxa and computing ancestral states allowed us to trace back the development of different mineralization patterns in deep time.

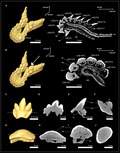

## Introduction

Modern sharks form a monophyletic group with rays and skates (Neoselachii *sensu *Compagno, 1977; Elasmobranchii *sensu* Maisey, 2012), with a fossil record extending back into the Early Permian (295 mya; Ivanov, 2005) and have developed a wide range of lifestyles and feeding strategies during their evolutionary history (Compagno, 1990a; Wilga et al. 2007). It is apparent that the continuous tooth replacement [polyphyodont dentition (Ifft & Zinn, 1948; Cappetta, 2012)] and the development of a variety of different tooth morphologies (Cappetta, 2012) are key features that allowed modern sharks to occupy a range of ecological roles, from ectoparasites like the cookie cutter shark *Isistius brasiliensis* (Papastamatiou et al. 2010) to apex predators such as the great white shark *Carcharodon carcharias* (Heupel et al. 2014). The morphology of shark teeth is thought to be related to their function, i.e. grasping, cutting or crushing prey (Frazzetta, 1988; Huber et al. 2009; Cappetta, 2012), although this assumption is challenged (Whitenack & Motta, 2010), and it is also used to resolve phylogenetic relations of extinct shark taxa (e.g. Kriwet et al. 2008; Cappetta, 2012). The role of tooth composition in feeding performance or as a taxonomic criterion has been tested for distinct taxa in the past, but remains ambiguous (Radinsky, 1961; Glickman, 1964; Whitenack et al. 2010; Moyer et al. 2015; Jambura et al. 2018; Martínez‐Pérez et al. 2018; Jambura et al. 2019).

Shark teeth can be subdivided into two external portions: the root, which is attached to a set of connective tissues enveloping the oral jaw cartilage surfaces (Peyer, 1968; Rasch et al. 2016; Smith et al. 2018) and the crown, which is used for prey capture during the feeding process (Compagno, 1988). The root consists of porous osteodentine, a kind of dentine which consists of dentinal osteons and interosteonal tissue, and superficially resembles osteonal bone (Radinsky, 1961; Berkovitz & Shellis, 2017). The tooth crown is composed of a dentinal core overlain by the hypermineralized enameloid, which is not homologous to the enamel of tetrapods. In shark teeth, the enameloid layer starts to mineralize before the dentine starts to form (in tetrapods it is vice versa) and the odontoblastic processes extend into the enameloid layer in shark teeth but are absent in tetrapod enamel (Peyer, 1968; Kemp, 1999). The dentinal core can consist of orthodentine, osteodentine, or both. Orthodentine exhibits parallel, branching tubules that were originally described to surround a hollow pulp cavity (Radinsky, 1961; Smith & Sansom, 2000), but which can also surround a dentinal core of osteodentine in shark teeth (Jambura et al. 2018)). Originally, two histological patterns were distinguished in shark teeth, the orthodont histotype (presence of a hollow pulp cavity) and the osteodont histotype [the pulp cavity is replaced by osteodentine that extends from the root (Tomes, 1876; Glickman, 1964; Compagno, 1988; Moyer et al. 2015; Schnetz et al. 2016; Martínez‐Pérez et al. 2018)]. More recent studies, however, defined three histotypes, depending on the presence or absence of orthodentine and osteodentine in the crown: (i) orthodont teeth, which have a central hollow pulp cavity, encapsulated by a substantial layer of orthodentine; (ii) pseudoosteodont teeth, in which osteodentine intrudes from the root into the hollow pulp cavity, which in fully mineralized teeth is replaced by an osteodont core that is surrounded by orthodentine; and (iii) osteodont teeth, in which no orthodentine is developed, but the complete dentinal core of the crown consists of osteodentine and replaces the hollow pulp cavity (Jambura et al. 2018; Jambura et al. 2019).

Two well‐examined shark groups in regard to their tooth histology are carcharhiniform and lamniform sharks. With the exception of the snaggletooth shark *Hemipristis elongata*, which has pseudoosteodont teeth, carcharhiniform sharks display the orthodont histotype (Compagno, 1973; Compagno, 1988; Herman et al. 1991; Herman et al. 2003; Jambura et al. 2018). In contrast, the sister group, lamniform sharks, exhibit the osteodont tooth histology, except for the basking shark *Cetorhinus maximus*, which also has pseudoosteodont teeth (Moyer et al. 2015; Schnetz et al. 2016; Jambura et al. 2018; Jambura et al. 2019). Studies on the histology of sharks of the orders Hexanchiformes (Herman et al. 2003), Squaliformes (Herman et al. 2003), Squatiniformes (Herman et al. 1992) and Orectolobiformes (Herman et al. 1992) were conducted and assigned to one of the two original tooth histotypes, overlooking the pseudoosteodont tooth histotype. However, in many cases, the description of dentine structures lacks essential information (e.g. the presence or absence of distinct dentine layers) or conflicts with the provided illustrations, preventing a sophisticated comparison of different histology patterns in modern sharks.

The aim of the present study was to provide an extensive overview of the distribution of tooth histology patterns in all extant orders of sharks and to identify the plesiomorphic condition for modern sharks. It is discussed, whether tooth histologies might bear a phylogenetic signal for modern sharks and/or if the tooth histology is instead linked to the function of the tooth (e.g. cutting, grasping, crushing).

## Materials and methods

### Material

To examine the distribution of the three tooth histotypes in modern sharks, 23 isolated teeth of 21 species from all nine extant shark orders and the extinct order †Synechodontiformes were examined. Ten teeth were from extant species, and 13 from extinct species (Mesozoic and Cenozoic). Additionally, three jaws were examined to investigate the tooth mineralization sequence in the orders Squatiniformes, Echinorhiniformes and Heterodontiformes. Extinct taxa are marked by a dagger preceding the taxon name. A list of all examined specimens and their repository is provided in Table S1.

### Computed tomography scanning and imaging

Tooth histology was examined non‐destructively with X‐ray tomographic imaging. Jaws and teeth of different sizes were scanned using three different micro‐computed tomography (CT) devices: SkyScan1173 micro‐CT device (Bruker, Kontich, Belgium) at the Department of Palaeontology (University of Vienna); Xradia MicroXCT‐system (Zeiss, Oberkochen, Germany) at the Department of Theoretical Biology (University of Vienna); and VISCOM X8060 NDT X‐ray (Viscom AG, Hannover, Germany) at the Department of Evolutionary Anthropology (University of Vienna). The applied device and settings for each specimen are summarized in the Table S2. The generated slice file stacks were loaded into the Amira software package (version 5.4.5; FEI Visualization Sciences Group, Hillsboro, OR, USA) to create isosurfaces and virtual sections through different planes of the examined teeth to investigate their internal anatomy. All raw data are stored on servers in the Department of Palaeontology (University of Vienna). Figures of the resulting two‐dimensional images were finalized with Adobe Photoshop CS6 (version 13.0; Adobe Systems, San José, CA, USA) in regard to editing colour balance, contrast and labelling.

### Tooth terminology

Tooth files and teeth with known positions within the jaw were labelled according to a modified version of Moyer et al. (2015): (i) the first letter indicates the position of the file right (R) or left (L) from the symphysis; (ii) the next two letters indicate the location of the tooth in either the lower jaw (MC = Meckel’s cartilage) or the upper jaw (PC = palatoquadrate cartilage); (iii) the last letter (T = tooth) is accompanied by a number and indicates the position of the tooth within the tooth file, starting from the most labial tooth (T1).

### Ancestral state reconstruction

A phylogenetic tree with 31 shark genera with known tooth histologies was built based on the NADH2 sequences used in the phylogeny of Naylor et al. (2012). The thornback ray *Raja clavata* was used as an outgroup to establish plesiomorphic trait conditions. The generated tree was a strongly pruned version of the original tree, which caused a heavily altered topology. To counteract these changes, the relationships between the used taxa were constrained to represent the original topology by running a maximum likelihood analysis under a backbone constraint (following the topology of Naylor et al. 2012) in the program RaxML (Stamatakis, 2014) in the CIPRES web portal (Miller et al. 2010). Note that the relationships between the clades Echinorhiniformes and Squatiniformes + Pristiophoriformes could not be resolved. This might be the result of the heavy pruning of these clades whose relationships were not well supported in the original tree either and, according to the authors, needed further exploration. In contrast to the original tree of Naylor et al. (2012) in which the genus *Alopias* did not represent a monophyletic group, we decided to treat this clade as a monophyly, since this is supported by previous molecular analyses based on cytochrome b (Martin & Naylor, 1997)) and whole mitochondrial genomes (Doane et al. 2018), as well as morphological analyses (Compagno, 1990b; Shimada, 2005). The topology of the resulting tree was used for stochastic character mapping with the make.simmap function in phytools (Revell, 2012) to perform ancestral state reconstructions of the three different tooth histotypes. The data for the tooth histology of the 31 shark genera were retrieved from the present study and the literature (Herman et al. 1992; Jambura et al. 2018; Jambura et al. 2019). In addition to the three histotypes (orthodont, osteodont and pseudoosteodont), the orthodont tooth histotype in *Raja* was differentiated from the orthodont tooth histology in sharks, since it shows significant differences from the latter group (i.e. orthodentine is not restricted to the crown, but makes up the whole tooth, with no osteodentine being present). To highlight this difference, the orthodont histology in rays was labelled as ‘regular orthodont’. The final tree was edited in FigTree (version 1.4.4) and Photoshop CS6 (version 13.0, Adobe Systems).

## Results

The tooth histotype of 23 species of all nine extant shark orders and the alleged stem group †Synechodontiformes was examined using micro‐CT imaging. Virtual sections of the CT‐based model of the teeth revealed the presence of a hypermineralized tissue (enameloid) covering the tooth crown, and one or two types of dentine: orthodentine and osteodentine. Different densities of mineralized structures result in different greyscales in the CT images. In the CT images of this study, enameloid is depicted in white, while dentine is grey. The two types of dentine can be distinguished from each other through structural differences. Orthodentine is made of parallel tubules that are not detectable with our imaging technique, giving it a dense appearance in the CT images shown in the present study without canals or pores. Orthodentine was present in the crown of all examined sharks, except for members of the order Lamniformes, whose teeth are only composed of osteodentine. Osteodentine is made up of dentinal osteons and interosteonal tissue, which are detectable in the CT images and result in its spongious appearance. Osteodentine is present in the tooth roots of all examined species (except for *Pristiophorus nudipinnis*) and might intrude into the pulp cavity of the crown to a varying extent. The tooth composition of each species is described within the section of its corresponding order.

### †Synechodontiformes

**Figure 1:**
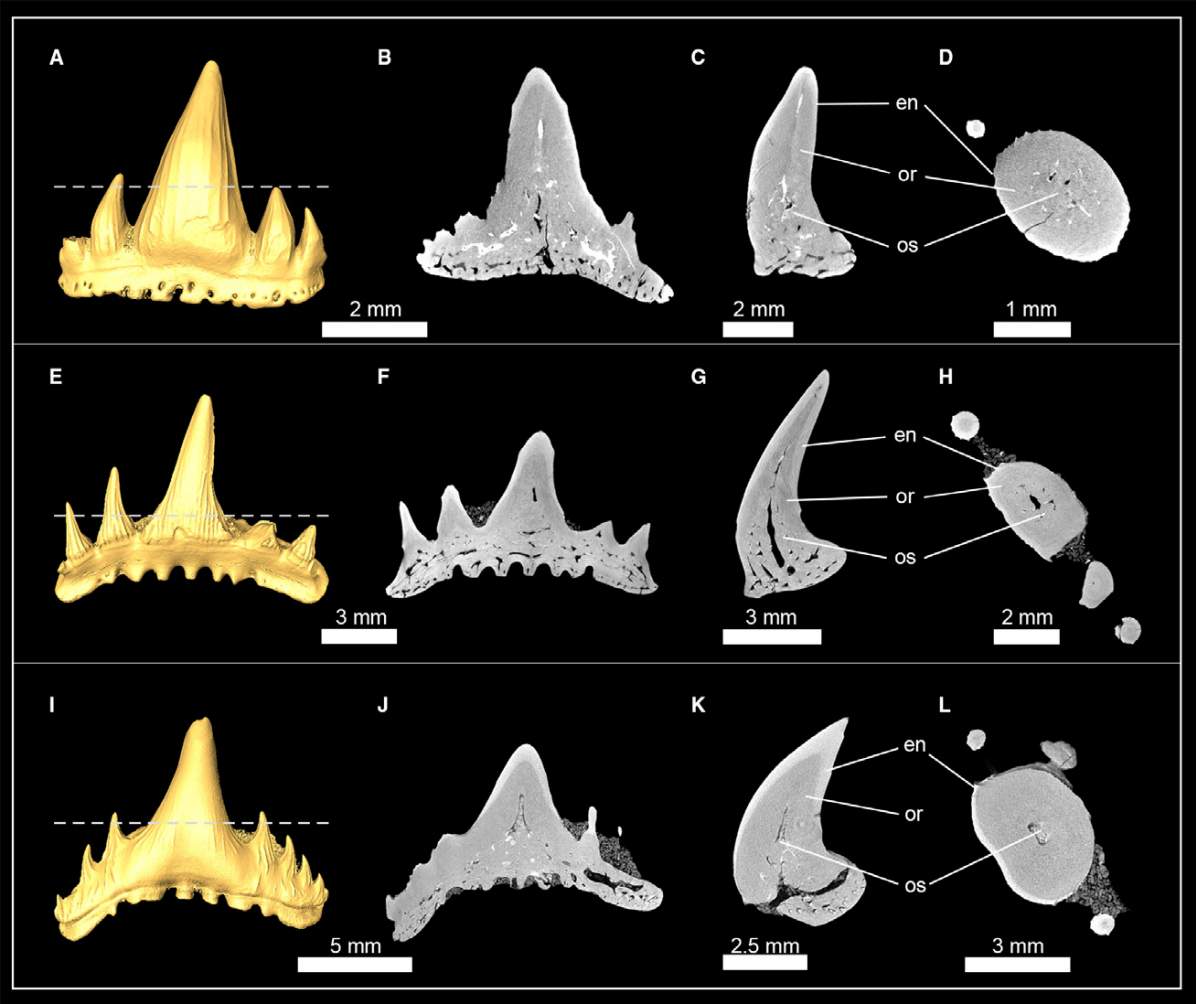
Isosurfaces and virtual tooth sections of the synechodontiform sharks †*Rhompaiodon minor* (EMRG‐Chond‐T‐40) (A), (EMRG‐Chond‐T‐41) (B–D), †*Paraorthacodus* sp. (SMNS‐87088) (E–H) and †*Synechodus* sp. (SMNS‐87099) (I–L). Tooth sections are in frontal (B,F,J), sagittal (C,G,K) and axial view (D,H,L). Dashed lines indicate where the plane of the axial tooth sections lie. en, enameloid; or, orthodentine; os, osteodentine.

The phylogenetic position, monophyly and taxonomy of synechodontiform sharks are still debated. In the present study we follow Klug (2010) and consider synechodontiform sharks a monophyletic group of elasmobranch stem‐group representatives. Three genera of synechodontiform sharks were examined: †*Synechodus* sp. (†Palaeospinacidae), †*Rhomphaiodon minor* (incertae familiae), and †*Paraorthacodus* sp. (†Paraorthacodontidae). All three species share similar tooth histologies (Fig. 1): underneath the enameloid is a thick layer of orthodentine that encapsulates the pulp cavity. Osteodentine from the root invades the pulp cavity basally and fills most of it in functional teeth, so that the hollow pulp cavity is reduced to a narrow central canal that extends from the root towards the apex of the crown. This canal is surrounded by dentinal osteons in †*R. minor* (Fig. 1A–D) and †*Paraorthacodus* sp. (Fig. 1E–H), which was not the case in †*Synechodus* sp. (Fig. 1I–L). In all three species, osteon cavities were secondarily filled with highly mineralized material to different degrees, which can be distinguished from dentine by its higher mass density; this results in a higher X‐ray absorption in the CT scans. The infilling of the pulp cavity with osteodentine and the presence of orthodentine represent the pseudoosteodont tooth histotype, which seemingly is common for synechodontiform sharks.

### Sharks of the superorder Squalomorphii

#### Hexanchiformes (frilled and cow sharks)

**Figure 2:**
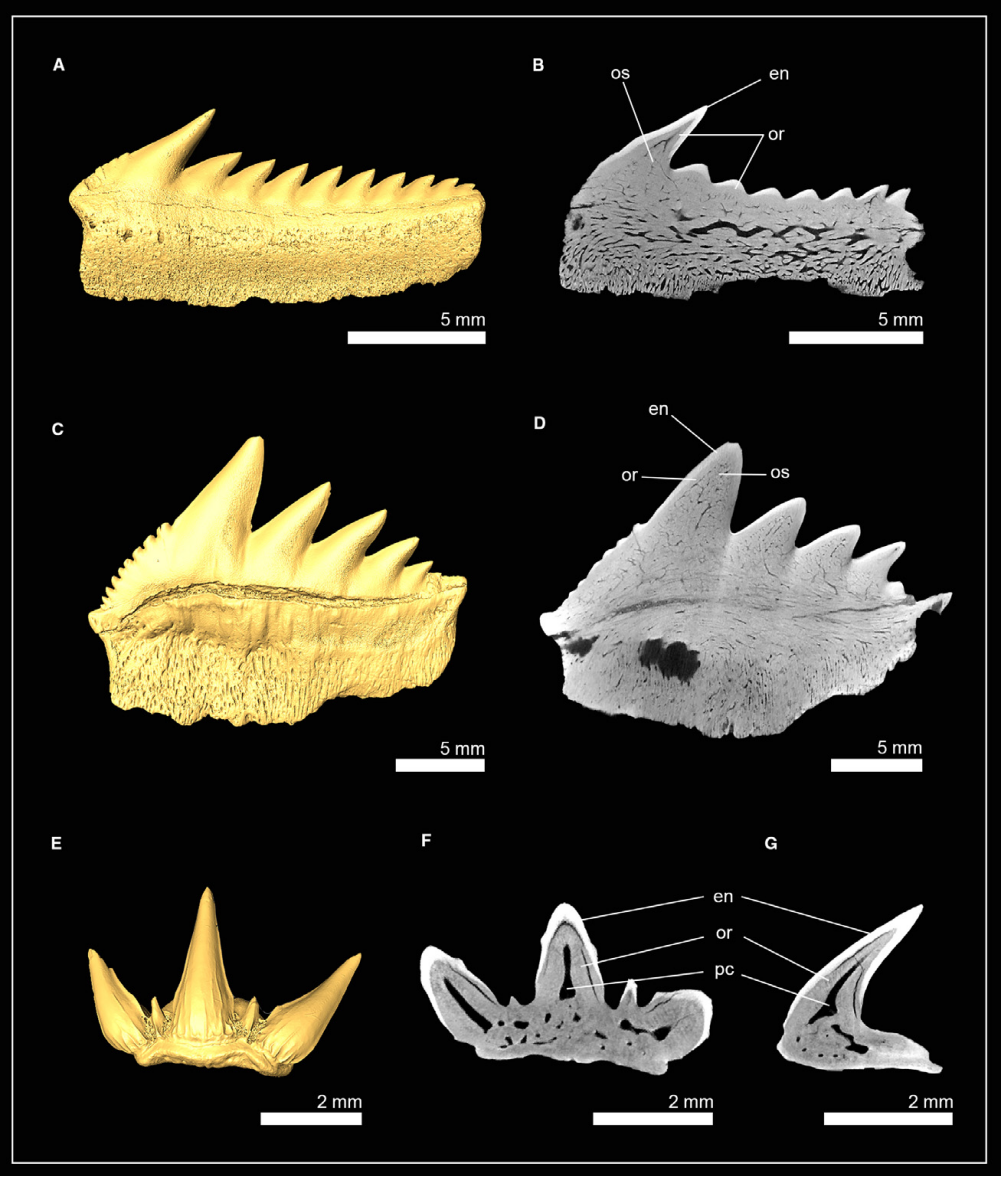
Isosurface and virtual tooth sections of hexanchiform sharks. (A,B) †*Hexanchus microdon* (EMRG‐Chond‐T‐38; Hexanchidae, tooth section in frontal view). (C,D) †*Notorynchus kempi* (NHM_2006z0274/0001; Hexanchidae, tooth section in frontal view). (E) *Chlamydoselachus anguineus* [EMRG‐Chond‐T‐66; Chlamydoselachidae, tooth section in frontal (F) and sagittal view (G)]. en, enameloid; or, orthodentine; os, osteodentine; pc, pulp cavity.

Tooth histology of three genera (*Hexanchus*, *Notorynchus* and *Chlamydoselachus*) of both hexanchiform families was examined using micro‐CT imaging (Fig. 2). In †*Hexanchus microdon* (Hexanchidae), the osteon network in the root consists of relatively coarse osteons, whereas osteons within the tooth crown are finer. Most of the pulp cavity in the crown is infilled by osteodentine. In the apex of the crown a narrow hollow canal is what remains of the former pulp cavity. Both the hollow canal of the pulp cavity and the osteodentine core are encapsulated by a prominent layer of orthodentine (Fig. 2A).

In †*Notorynchus kempi* the pulp cavity is filled with osteodentine, leaving no hollow pulp cavity or canals. The dentinal osteon network in both the root and the crown consists of rather delicate branches. Orthodentine is present between the enameloid and the osteodont core of the crown. Both representatives of the family Hexanchidae therefore have the pseudoosteodont tooth histotype (Fig. 2B).

In the frilled shark *Chlamydoselachus anguineus* (Chlamydoselachidae), osteodentine is restricted to the roots and is not invading the hollow pulp cavity of the crown, which is retained in all three cusps. The only dentine present in the tooth crown of *Chlamydoselachus* is orthodentine; therefore, *C. anguineus* displays the orthodont histotype (Fig. 2D,E).

##### Squaliformes (dogfish sharks)

**Figure 3:**
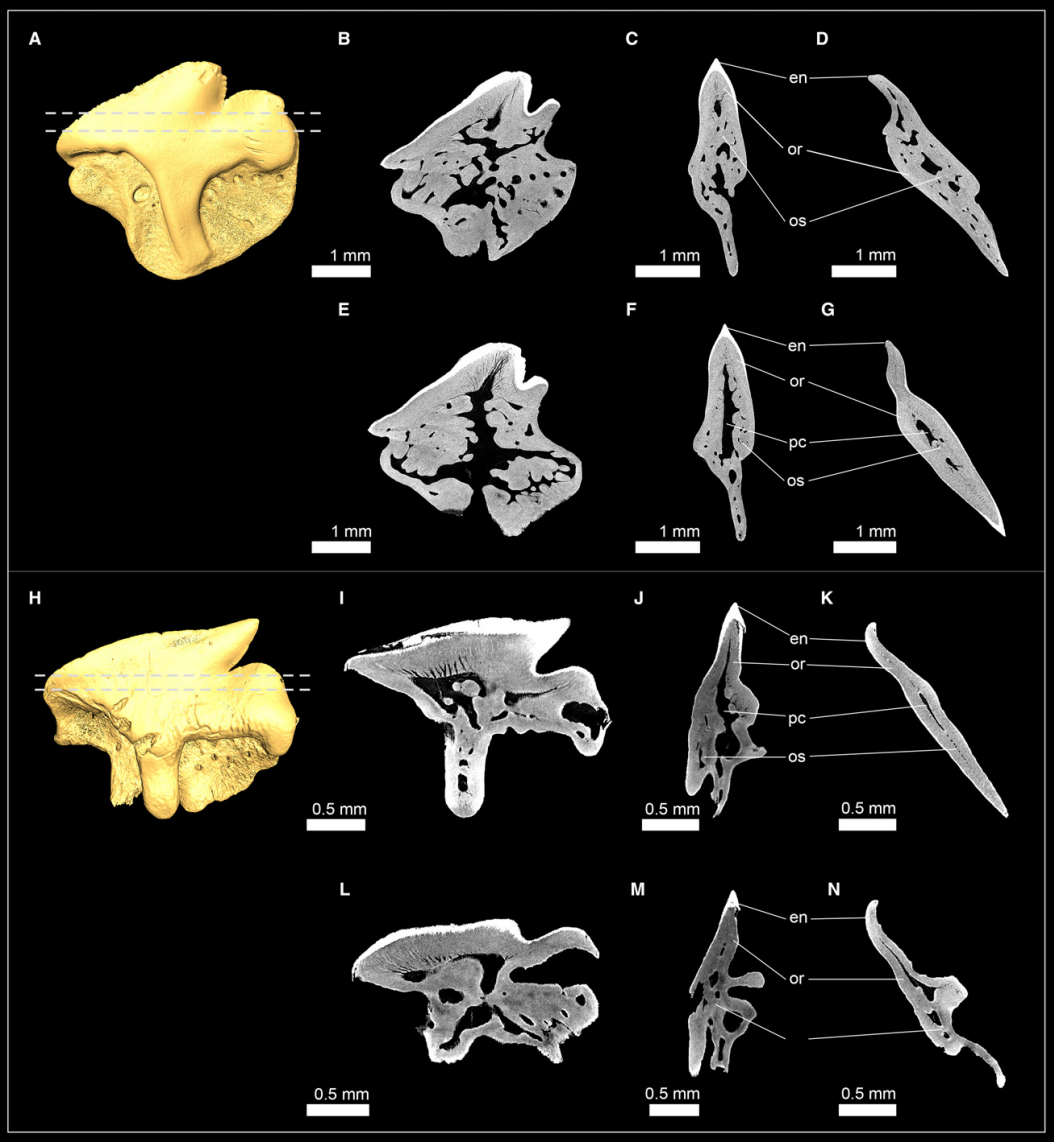
Isosurfaces and virtual tooth sections of the squaliform sharks *Squalus acanthias* (EMRG‐Chond‐T‐63) (A–G) and *Centrophorus granulosus* (EMRG‐Chond‐T‐62) (H–N). Virtual sections go through the tooth in different angles: frontal (B,E,I,L), sagittal (C,F,J,M) and axial view (D,G,K,N). Dashed lines indicate where the plane of the axial tooth sections lie. en, enameloid; or, orthodentine; os, osteodentine; pc, pulp cavity.

High resolution micro‐CT images of the teeth RMC3T1 of the spiny dogfish *Squalus acanthias* (Squalidae) and RMC4T1 of the gulper shark *Centrophorus granulosus* (Centrophoridae) reveal a modified tooth mineralization pattern in this group (Fig. 3). In *C. granulosus*, a thick layer of orthodentine lies under the enameloid and encapsulates the apical part of the hollow pulp cavity (Fig. 3B,C,E,F). The pulp cavity extends basally into the root and is surrounded by osteodentine. Sagittal tooth sections show that osteodentine is not restricted to the root but also intrudes into the crown and replaces parts of the hollow pulp cavity there, resulting in a reduced cavity, which is basally surrounded by osteodentine. In contrast to other sharks, the osteodentine network in both the root and the crown consists of very coarse osteons. In the crown, osteodentine is encased by orthodentine and enameloid (Fig. 3C,F) and, although a reduced pulp cavity remains, represents the pseudoosteodont tooth histotype (Figure 3A–F).

*Squalus acanthias* displays a similar histology pattern, although the hollow pulp cavity is more prominent than in *C. granulosus* (Fig. 3I–N). Sagittal tooth sections still reveal a small portion of osteodentine in the crown and, therefore, represent the pseudoosteodont tooth histotype. It is important to note that the presence of a hollow pulp cavity, which is partly surrounded by orthodentine, demonstrates a variation of this histotype in this group.

#### Squatiniformes (angel sharks)

**Figure 4:**
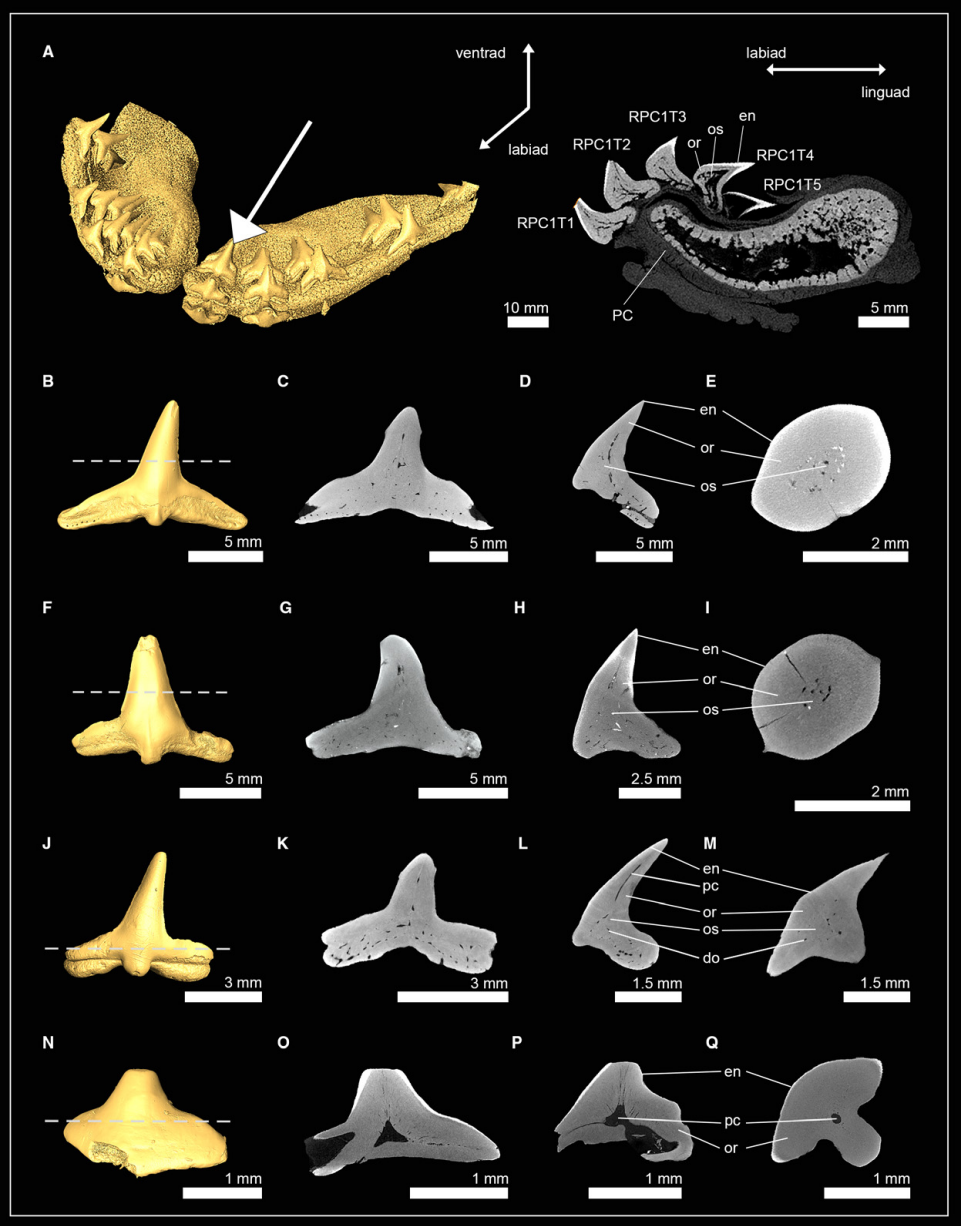
Isosurfaces and virtual sections of jaws and teeth of sharks of the orders Squatiniformes and Pristiophoriformes. (A) *Squatina squatina* (EMRG‐Chond‐J‐17). (B) †*Squatina subserrata* [NHM_1990/1487/0018a; tooth sections in (C) frontal view, (D) sagittal view and (E) axial view]. (F) †*Squatina angeloides* [EMRG‐Chond‐T‐68; tooth sections in (G) frontal view, (H), sagittal view (I) and axial view]. (J) †*Squatina prima* [EMRG‐Chond‐T‐69; tooth sections in (K) frontal view, (L), sagittal view (M) and axial view]. (N) *Pristiophorus nudipinnis* [EMRG‐Chond‐T‐61; tooth sections in (O) frontal view, (P), sagittal view (Q) and axial view]. Dashed lines indicate where the plane of the axial tooth sections lie. en, enameloid; or, orthodentine; os, osteodentine; pc, pulp cavity; PC, palatoquadrate cartilage.

Teeth of one extant and three extinct squatiniform sharks were examined: *Squatina squatina*; †*Squatina subserrata*; †*Squatina angeloides*; and †*Squatina prima*. In *S. squatina* each tooth file represents a developmental sequence and has four to five teeth in each of the four most anterior files (Fig. 4A). During tooth development, the enameloid is the first structure to mineralize and is already fully formed in the earliest detectable mineralization stage (RPC1T5). In the subsequent stage, RPC1T4, orthodentine forms underneath the enameloid and osteodentine in the root, which starts to invade the pulp cavity of the crown. Tooth formation is completed early, with the functional tooth (RPC1T1) and the two oldest replacement teeth (RPC1T2 and RPC1T3) already having fully filled pulp cavities. All four species showed the same tooth histotype: underneath the enameloid, a prominent layer of orthodentine surrounds a core of osteodentine, which replaced most of the hollow pulp cavity. Only a narrow hollow canal remains in the apex of the crown of the previous hollow pulp cavity (Fig. 4A–M). This is especially apparent in the tooth of †*Squatina prima*, in which only the basal part of the crown is filled with osteodentine, while the remaining pulp cavity is still present in the apex of the crown. The presence of both orthodentine and osteodentine within the crown of *Squatina* spp. demonstrates the presence of the pseudoosteodont tooth histotype in this group.

#### Pristiophoriformes (sawsharks)

The examination of the interior anatomy of a functional lower oral tooth (LMC1T1) of the shortnose sawshark *P. nudipinnis* revealed a highly modified tooth histology pattern for this species (Fig. 4N–Q). A hollow pulp cavity is retained in the crown and root of the tooth. From the hollow pulp cavity, vascular tubes spread out, with most of them being oriented towards the apex. These vascular tubes are highly ordered, in contrast to the dentinal osteons known from osteodentine, which are extending towards the enameloid more irregularly. This pulp cavity‐vascular tubes complex is surrounded by a very prominent layer of orthodentine, which makes up most of the tooth crown. No osteodentine could be identified within the root, in which the dentine has a very dense appearance, without dentinal osteons (orthodentine). This lack of osteodentine in the root, and the presence of a pulp cavity‐vascular tubes complex, which can neither unambiguously be identified as part of the orthodentine nor as modified dentinal osteons (and therefore osteodentine), demonstrate the presence of a highly modified tooth histology in *P. nudipinnis* which differs from the three known tooth histotypes.

#### Echinorhiniformes (bramble sharks)

**Figure 5:**
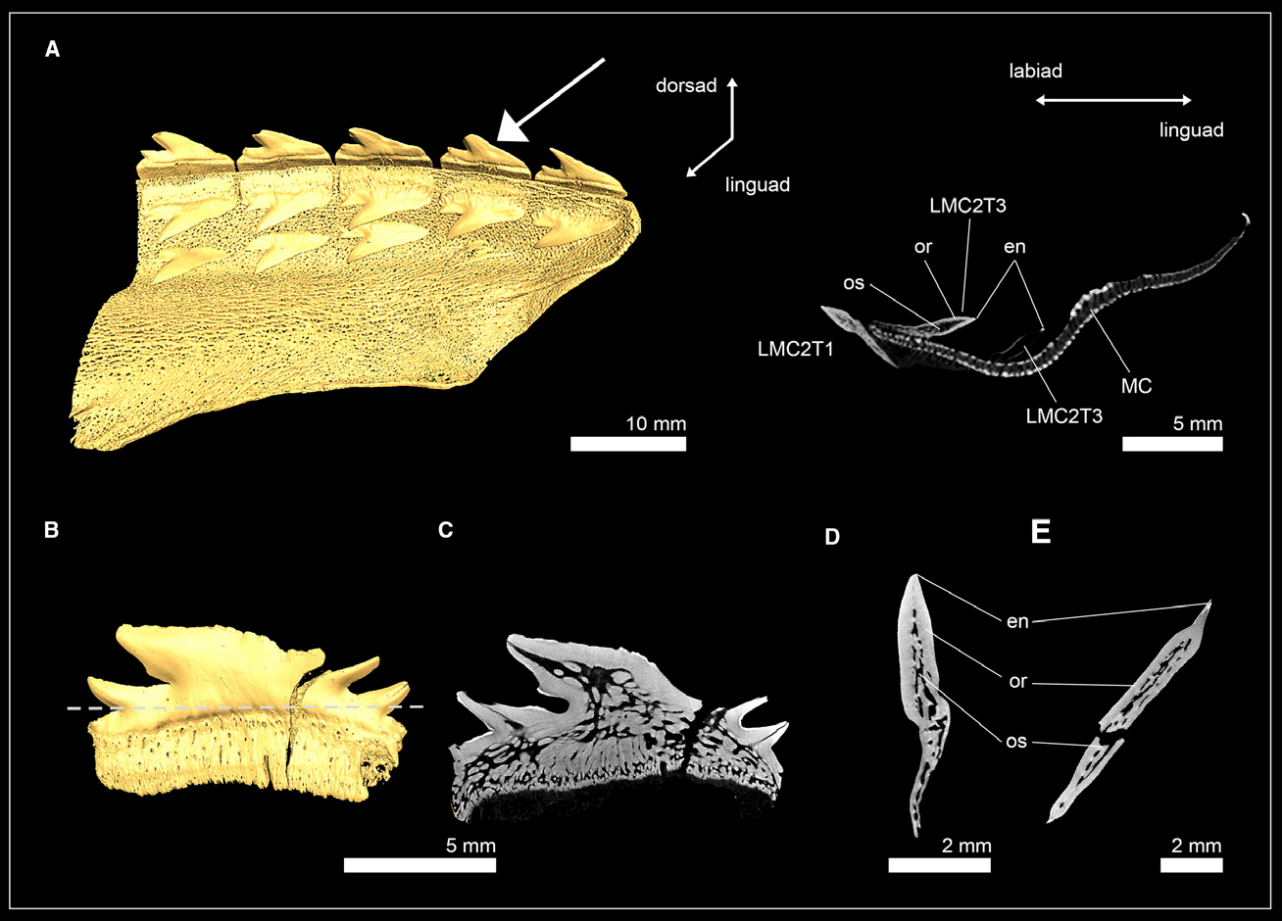
Isosurface and virtual sections of a jaw and an isolated tooth of two echinorhiniform sharks. (A) Virtual section of a tooth series from the lower jaw of *Echinorhinus brucus* (EMRG‐Chond‐J‐19). (B–E) Virtual sections of the isolated tooth of *Echinorhinus cookei* (EMRG‐Chond‐T‐64) in (C) frontal, (D) sagittal and (E) axial view. The dashed line indicates where the plane of the axial tooth section lies. en, enameloid; MC, Meckel’s cartilage; or, orthodentine; os, osteodentine.

High‐resolution micro‐CT images of tooth series of the lower jaw of the bramble shark *Echinorhinus brucus* and an isolated tooth of the prickly shark *Echinorhinus cookei* revealed the tooth histology pattern for this group (Fig. 5). *E. brucus* has tooth series consisting of three teeth: one functional tooth in erect position and two replacement teeth that are inverted, with their tips pointing lingually (except for the first row on each side next to the symphysis in which one functional and one replacement tooth form the tooth file). The youngest (most lingually situated) tooth (LMC2T3) only consists of enameloid. In the second replacement tooth, LMC2T3, a layer of orthodentine lies underneath the enameloid, and osteodentine starts to form in the roots and subsequently invades the hollow pulp cavity of the crown basally. In the functional tooth, LMC2T1, the hollow pulp cavity is fully filled with osteodentine (Fig. 5A). In both species the osteon network of the crown and the root consists of coarse osteons. Lateral cusplets show no sign of osteodentine intrusion and retain a thin hollow channel that is encapsulated by a thick layer of orthodentine. The presence of ortho‐ and osteodentine in the crown of both recent species display the pseudoosteodont tooth histology in this group.

### Sharks of the superorder Galeomorphii

#### Heterodontiformes (bullhead sharks)

**Figure 6:**
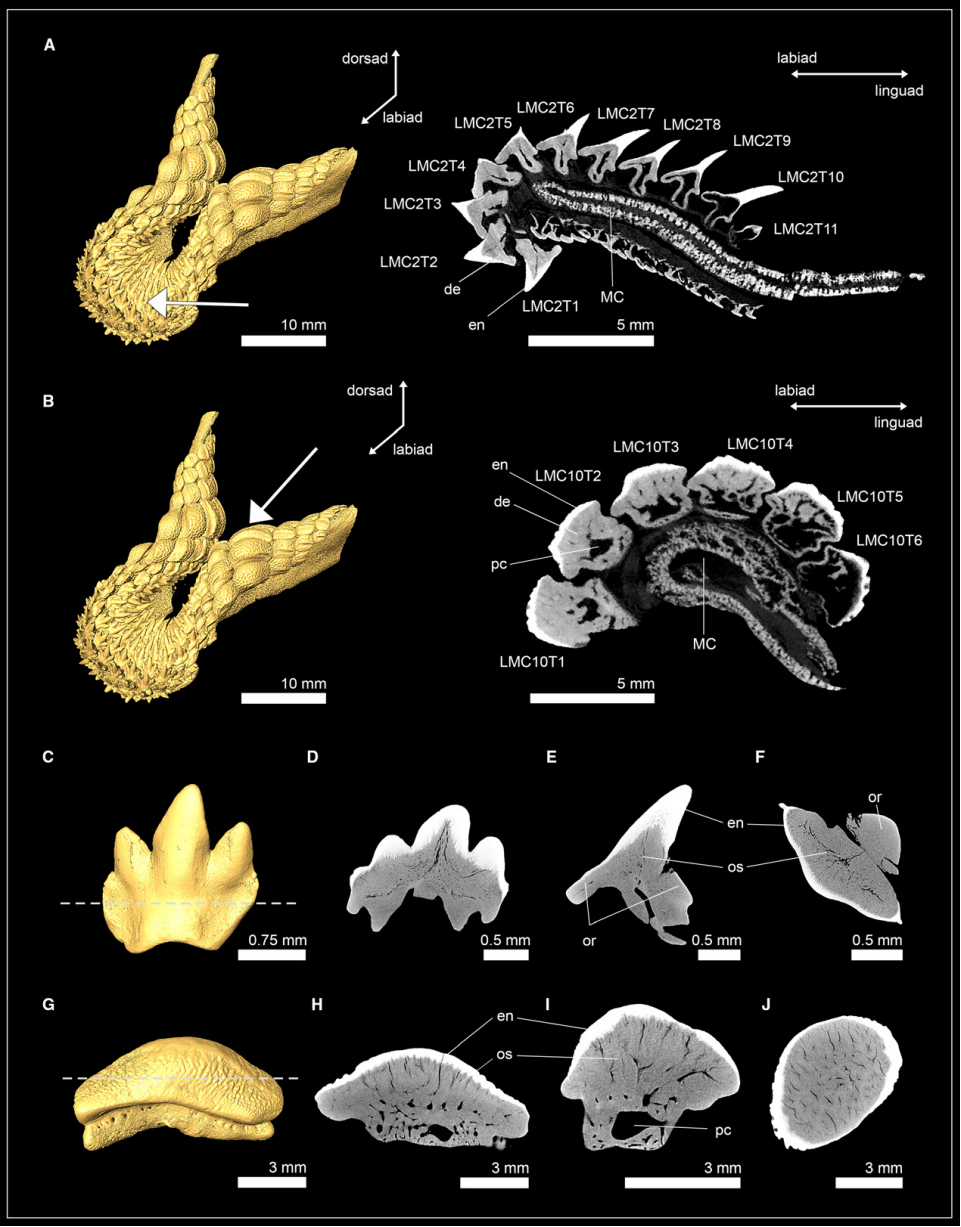
Isosurfaces and virtual sections of teeth from the heterodontiform shark *Heterodontus portusjacksoni*. (A) Anterior tooth series of the lower jaw (EMRG‐Chond‐J‐20). (B) Posterior tooth series of the lower jaw (EMRG‐Chond‐J‐20). (C) Isosurface of a molar tooth (EMRG‐Chond‐T65) and virtual sections in (D) frontal, (E) sagittal and (F) axial view. (G) Isosurface of an anterior tooth (EMRG‐Chond‐J‐20) and virtual sections in (H) frontal, (I) sagittal and (J) axial view. Dashed lines indicate where the plane of the axial tooth sections lie. de, dentine; en, enameloid; MC, Meckel’s cartilage; or, orthodentine; os, osteodentine; pc, pulp cavity.

The bullhead shark (Heterodontiformes) group is characterized by its very strong heterodonty and has small anterior teeth with cuspidate crowns and larger molariform teeth posteriorly that are not cuspidate. Micro‐CT slices through anterior and posterior tooth series (LMC2 and LMC10) were compiled to examine possible differences of the tooth mineralization sequence in the morphologically very different teeth of the Port Jackson shark *Heterodontus portusjacksoni* (Fig. 6A,B). The earliest developmental stage of the anterior teeth LMC2T11 only consists of enameloid. In the following developmental stages, dentine is forming and gradually filling the whole tooth (Fig. 6A). Virtual tooth sections of the isolated anterior tooth LMC2T1 show a pulp cavity that is filled with porous osteodentine and dentinal osteons reaching the enameloid at the apex of the crown. A compact layer of dentine is present on the labial and lingual sides at the base of the tooth, but not in the apex of the crown. The presence of orthodentine and osteodentine within the crown of the functional teeth are characteristic of the pseudoosteodont tooth histotype, but the replacement of orthodentine by osteodentine in the apex of the crown represents a modification (Fig. 6C–F). In the posterior tooth series LMC10, the youngest tooth LMC10T6 already exhibits enameloid and dentine. Dentine forms within the root and is detectable as blotches within the pulp cavity of the crown, but not as a compact layer underneath the enameloid, therefore, not representing orthodentine but osteodentine. During tooth development, no orthodentine is developed, but the blotches within the pulp cavity become denser and are traversed by osteons, which are coarse at the basis and become thinner and ramify close to the enameloid. Dentinal osteons are trending towards the enameloid and are to a considerable degree parallel to each other. A small hollow pulp cavity remains within the roots close to the opening of the median duct, but not within the crown (Fig. 6B,G–J). The presence of osteodentine and the absence of orthodentine are characteristic of the osteodont tooth histotype. Therefore, anterior and lateral (posterior) teeth of *H. portusjacksoni* follow different tooth mineralization pathways, resulting in pseudoosteodont anterior teeth with a reduced orthodont layer at the base of the tooth crown and the distal molariform teeth, in which orthodentine is replaced completely by osteodentine.

#### Orectolobiformes (carpet sharks)

**Figure 7:**
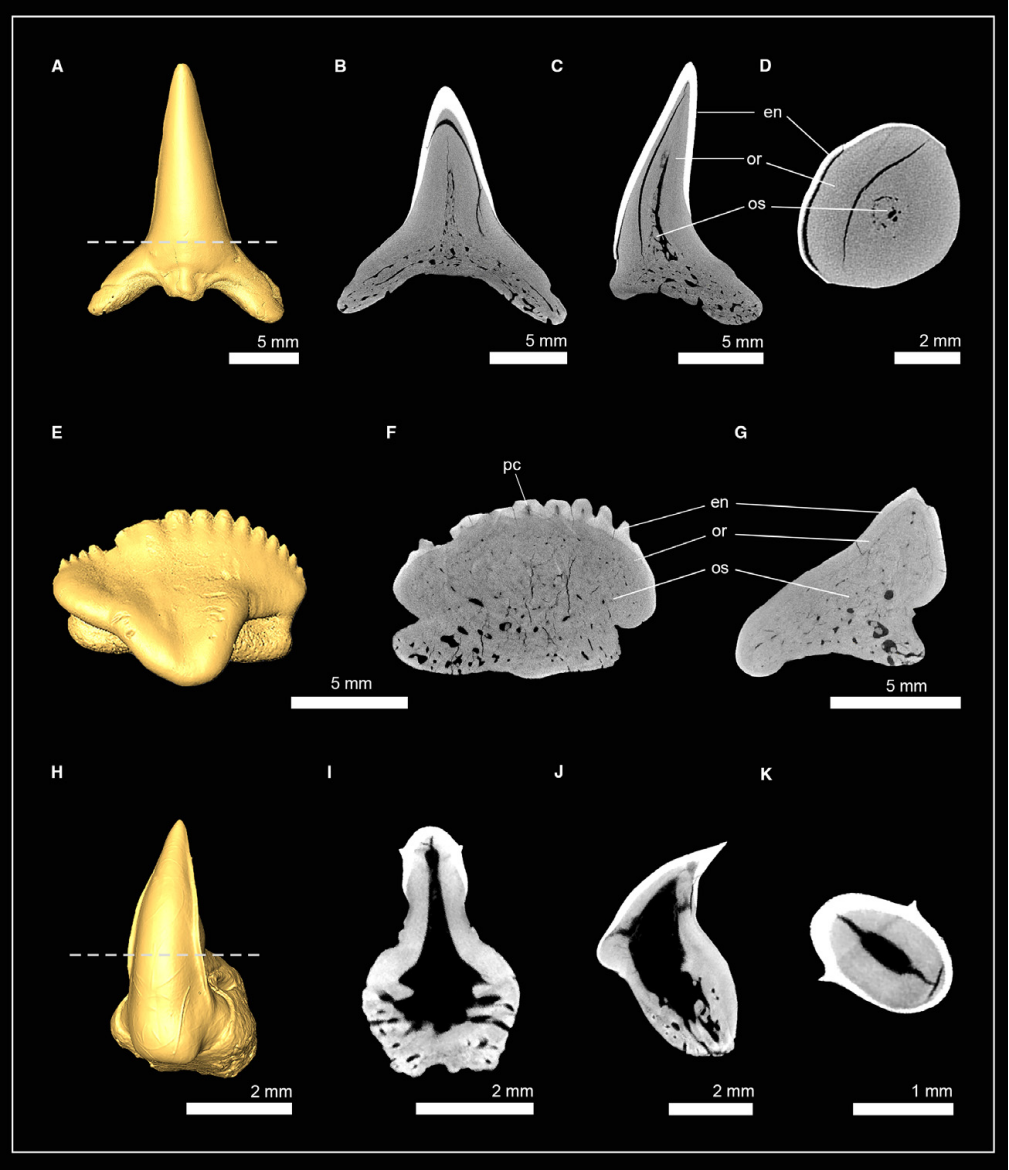
Isosurfaces and virtual sections of teeth of sharks from the order Orectolobiformes. (A) *Orectolobus maculatus* [EMRG‐Chond‐T‐67; Orectolobidae, tooth sections in (B) frontal view, (C) sagittal view, (D) axial view]. (E) †*Nebrius blanckenhorni* (NHM_1978/1966/0024a; Ginglymostomatidae); tooth sections in (F) frontal view and (G) sagittal view; (H) *Rhincodon typus* (Inv. no: 7‐714/RZ; Rhincodontidae); tooth sections in (I) frontal view, (J) sagittal view and (K) axial view. Dashed lines indicate where the plane of the axial tooth sections lie. en, enameloid; or, orthodentine; os, osteodentine; pc, pulp cavity.

Teeth of three orectolobiform shark species were examined: *Orectolobus maculatus* (Orectolobidae); †*Nebrius blanckenhorni* (Ginglymostomatidae); and *Rhincodon typus* (Rhincodontidae). The tooth crown of the spotted wobbegong *O. maculatus* is composed of a thick layer of orthodentine underneath the enameloid, which encapsulates a narrow pulp cavity. The pulp cavity is partly filled by osteodentine, which intrudes from the root and only a narrow hollow canal remains in the apex of the crown (Fig. 7A–D).

In †*N. blanckenhorni*, the orthodentine layer is less prominent than in *O. maculatus*. The pulp cavity is enlarged and completely filled with osteodentine. The serrations of the tooth cutting edges only consist of orthodentine, and each serration has a hollow cavity. The presence of ortho‐ and osteodentine in the crowns of both species constitute the pseudoosteodont tooth histotype (Fig. 7E–G).

Teeth of the whale shark *R. typus* have an orthodentine layer that surrounds an enlarged hollow pulp cavity. Osteodentine is restricted to the roots and is not intruding into the crown; therefore, *R*. *typus* teeth exhibit the orthodont tooth histotype (Fig. 7H–K).

#### Carcharhiniformes (ground sharks)

**Figure 8:**
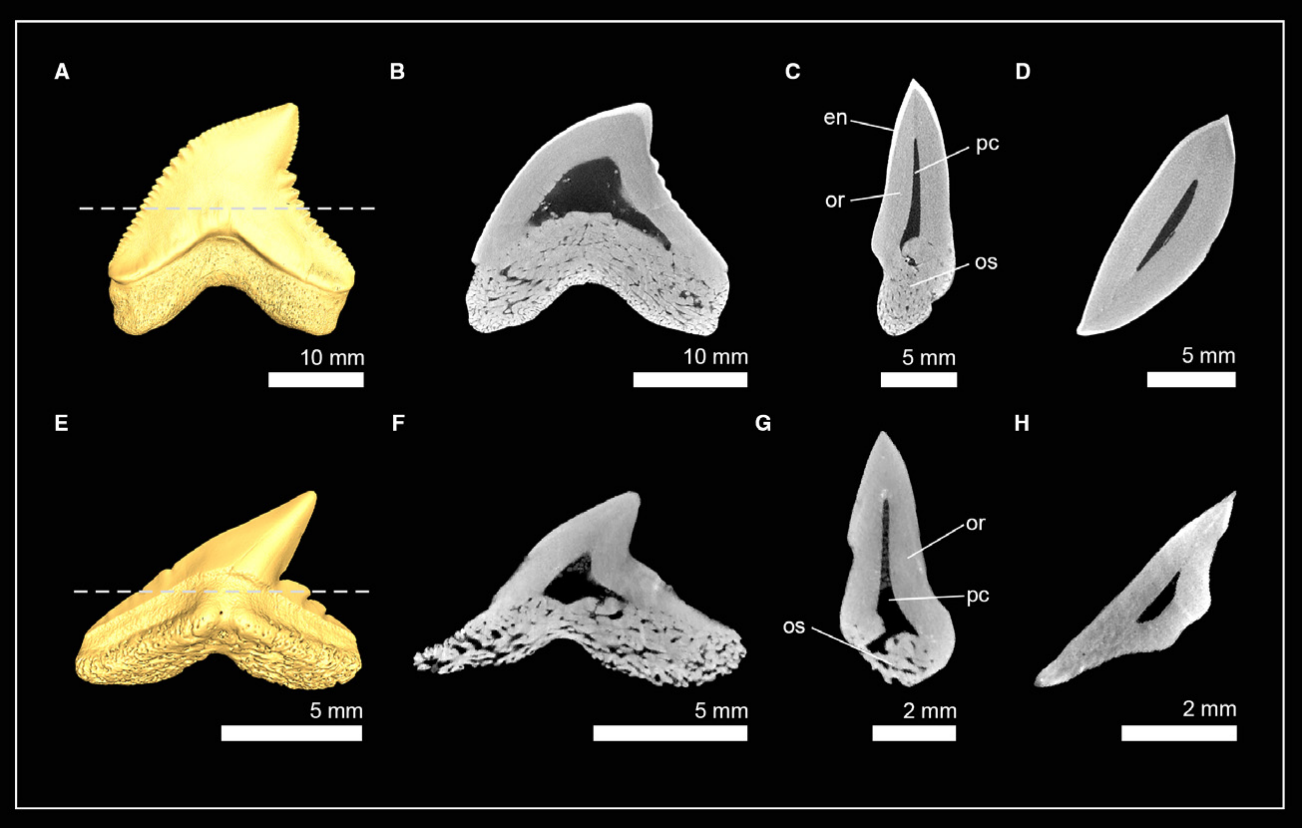
Isosurfaces and virtual tooth sections of teeth of sharks from the order Carcharhiniformes. (A) †*Galeocerdo mayumbensis* (Inv. no: 7‐713; Carcharhinidae); tooth sections in (B) frontal view, (C) sagittal view and (D) axial view. (E) †*Physogaleus* sp. (Inv. no: 7‐716; Carcharhinidae); tooth sections in (F) frontal view, (G) sagittal view and (H) axial view. Dashed lines indicate where the plane of the axial tooth sections lie. en, enameloid; or, orthodentine; os, osteodentine; pc, pulp cavity.

Two closely related fossil taxa of carcharhiniform sharks were examined: †*Galeocerdo mayumbensis* and †*Physogaleus* sp. Both species display a hollow pulp cavity in the tooth crown that is encapsulated by a prominent layer of compact dentine, orthodentine, and both contain very dense minerals within the pulp cavity, which infiltrated the tooth during fossilization, and can be separated from dentine by its structure and density (greyscale) (Fig. 8). Enameloid was detectable in †*G. mayumbensis*, but not in †*Physogaleus* sp., which could be caused by diagenetic processes that altered the chemical constitution (and density) of the enameloid (see also Jambura et al. 2019). Both species display the orthodont histotype of most carcharhiniformn sharks except for *Hemiprists* (Jambura et al. 2018).

#### Lamniformes (mackerel sharks)

**Figure 9:**
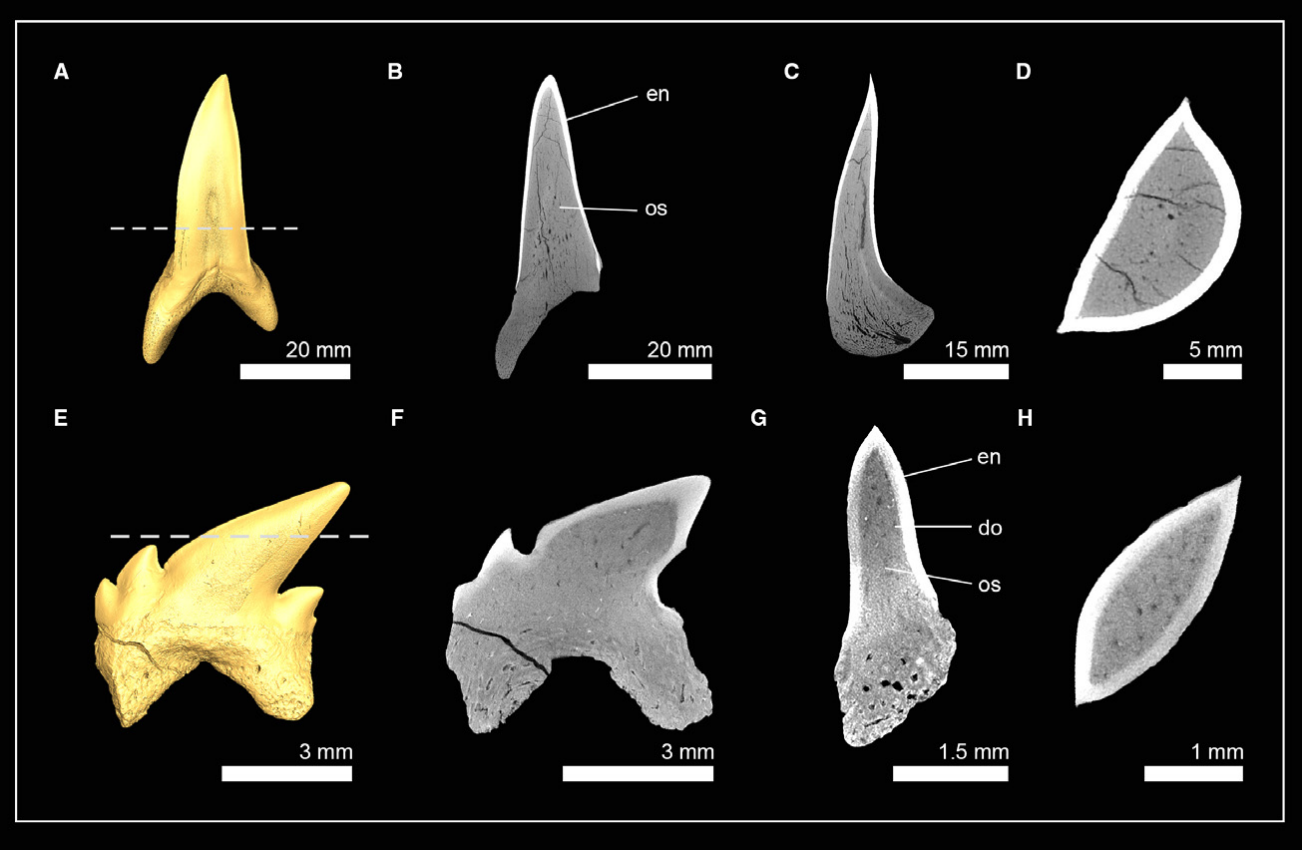
Isosurfaces and virtual tooth sections of teeth of sharks from the order Lamniformes. (A) *Isurus paucus* (Inv. no: 7‐715/RZ; Lamnidae); tooth sections in (B) frontal view, (C) sagittal view and, (D) axial view. (E) †*Haimirichia amonensis* (Inv. no: 7‐09; †Haimirichiidae); tooth sections in (F) frontal view, (G) sagittal view and (H) axial view. Dashed lines indicate where the plane of the axial tooth sections lie. do, dental osteons; en, enameloid; os, osteodentine.

Teeth of the longfin mako shark *Isurus paucus* (Lamnidae) and the extinct lamniform shark †*Haimirichia amonensis* (†Haimirichiidae) consist of two components: a hypermineralized outer tissue layer, the enameloid, and a core of dentine (Fig. 9). The dentinal core is completely traversed by dentinal osteons that give it a bone‐like appearance. Therefore, the present dentine is osteodentine. A second layer of dentine, orthodentine, between osteodentine and enameloid is not developed in this group. *I. paucus* and †*H. amonensis* therefore exhibit exclusively the osteodont tooth histotype.

## Discussion

### Ancestral states of the tooth histologies in modern sharks

Despite extensive investigation of shark tooth histology in the orders Carcharhiniformes (Compagno, 1988; Herman et al. 2003; Jambura et al. 2018) and Lamniformes (Moyer et al. 2015; Schnetz et al. 2016; Jambura et al. 2018; Jambura et al. 2019), a comprehensive comparative study considering the distribution of different tooth histologies among modern sharks has not been conducted before. In the present study, tooth histologies for sharks of all nine extant orders and the putative stem group †Synechodontiformes were examined.

**Figure 10:**
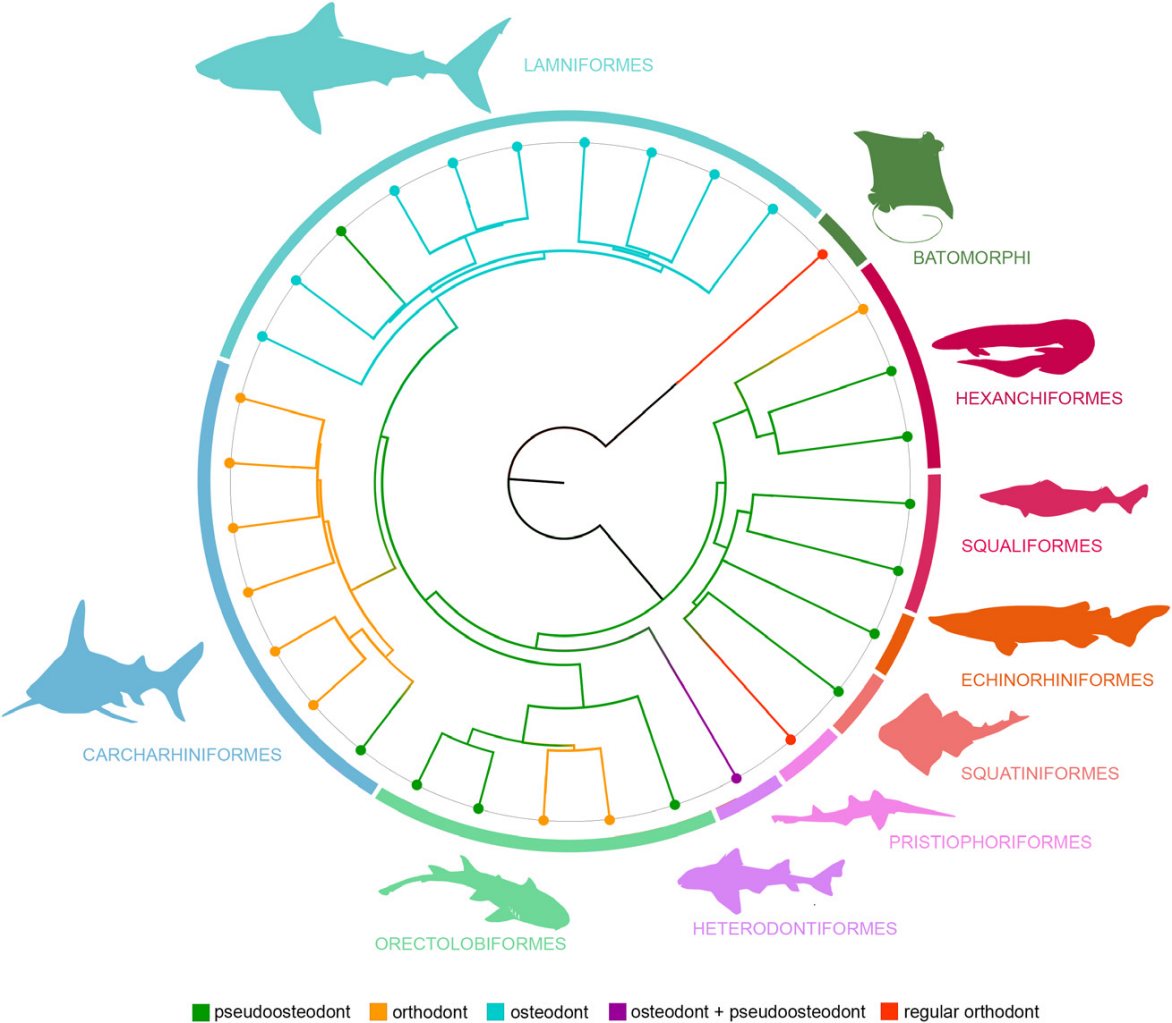
Ancestral state reconstruction for the tooth mineralization patterns in modern sharks. The phylogenetic tree is based on whole mtDNA sequences. Ancestral states were calculated from 100 stochastic mappings for the three defined histotypes (orthodonty, osteodonty, pseudoosteodonty) and the two modified patterns which were found in *Heterodontus portusjacksoni* (osteodont molar teeth, pseudoosteodont anterior teeth) and *Pristiophorus nudipinnis* (a state only known from rays, here referred to as ‘regular orthodont’).

Our findings demonstrate that the pseudoosteodont tooth histotype (the presence of orthodentine and osteodentine in the tooth crown) is the most common tooth histology in modern sharks. Except for the frilled shark *C. anguineus* and the sawshark *P. nudipinnis*, all sharks of the superorder Squalomorphii exhibit the pseudoosteodont tooth histotype. Within the Galeomorphii, the pseudoosteodont tooth histotype is represented in each order, but to a lesser extent than in sharks of the superorder Squalomorphii. In the two most derived galeomorph shark orders, Carcharhiniformes and Lamniformes, only a single extant species in each group exhibits this histotype: *H. elongata* [Carcharhiniformes (Compagno, 1973; Compagno, 1988; Jambura et al. 2018)] and *C. maximus* (Lamniformes), respectively (Jambura et al. 2019). All other carcharhiniform sharks are known to exhibit the orthodont histotype (Compagno, 1988; Hovestadt & Hovestadt‐Euler, 1993; Jambura et al. 2018), while the remaining lamniform sharks exhibit the osteodont tooth histotype, which is known exclusively for this group (Jambura et al. 2019). The ancestral state analysis suggests that the pseudoosteodont tooth histotype is the plesiomorphic condition for all modern sharks (Fig. 10, Fig. S1). This is further supported by our data on the tooth histology of synechodontiform sharks, alleged stem‐group elasmobranchs (Klug, 2010), which also is characterized by the pseudoosteodont tooth histotype. Surprisingly, in the sister group of modern sharks, the batomorphs (rays and skates), members of the most basal extant order Rajiformes have teeth that only comprise orthodentine, but not osteodentine (Herman et al. 1994). It is important to note, that the orthodont histotype in rays is different from most orthodont sharks (except for *Pristiophorus*) in lacking osteodentine in the root. This was already described by Peyer (1968) who stated that teeth of *Raja* have an ‘extraordinarily regular orthodentine structure’. We therefore refer to this histotype found in many rays and in *Pristiophorus* as ‘regular orthodont’. Since synechodontiform sharks are thought to be stem‐group representatives of both sharks and batomorphs (rays and skates), a transition from a pseudoosteodont tooth histotype to a regular orthodont histotype must have occurred very early during the evolution of batomorphs.

The phylogenetic relevance of different tooth histology patterns in chondrichthyans has been discussed in previous works, but clear conclusive interpretations are still lacking (Radinsky, 1961; Glickman, 1964; Compagno, 1973; Blazejowski, 2004; Maisey et al. 2004; Jambura et al. 2018). Our analyses support a clear trend among all sharks of the same order to show similar histological patterns; the only known exceptions are orectolobiform sharks, the frilled shark *Chlamydoselachus* [orthodont rather than pseudoosteodont (Goto & Hashimoto, 1976)], the snaggletooth shark *Hemipristis* [pseudoosteodont instead of orthodont (Compagno, 1988; Jambura et al. 2018)], and the basking shark *Cetorhinus* [pseudoosteodont instead of osteodont (Jambura et al. 2019)]. The underlying reasons for these variations cannot be explained solely within a phylogenetic, or a functional context, and therefore remain unresolved at present.

The pseudoosteodont tooth histotype is not only the most likely ancestral state for modern sharks, but is also the predominant histotype among modern sharks. Nevertheless, it is important to note that a high degree of variation can be identified: in pseudoosteodont teeth, orthodentine can be present as a very thick and prominent layer (e.g. *O. maculatus*, †*Synechodus* sp.), a thin layer (†*N. kempi,* †*Nebrius blankenhorni*), or reduced to the base of the tooth crown (anterior teeth of *H. portusjacksoni*).

The dentinal osteons of the osteodentine in the root and the crown can also show variations. Echinorhiniform sharks for instance have a very coarse canal system, while in other groups (e.g. Hexanchidae) the dentinal osteons appear much more delicate and the interosteonal tissue denser. Also, the degree to which the pulp cavity is filled with osteodentine may vary among taxa. While in most taxa the whole pulp cavity of the teeth is filled with osteodentine, the teeth of sharks of the order †Synechodontiformes display remnants of a hollow canal in the apex of the crown.

Another group showing a high degree of variation are the squaliform sharks, in which the hollow pulp cavity extends basally into the root but is partly replaced by osteodentine in the crown, which agrees with observations by Moyer & Bemis (2016). Herman et al. (2003) described a reticulated system of coarse osteons in squaliform shark teeth, which therefore confirms the presence of osteodentine in the crown and the pseudoosteodont tooth histotype in this group. However, these variations indicate that the histotype concept in its current state with three categories (orthodonty, osteodonty and pseudoosteodonty) is an oversimplification of the plethora of different histology patterns that can be observed in modern sharks and thus may not be sufficient to draw clear conclusions about its phylogenetic relevance.

Recently, a study using synchrotron tomography images to reconstruct the vascular system of teeth of the two oldest known sharks †*Leonodus carlsi* and †*Celtiberina maderi* (Martínez‐Pérez et al. 2018) showed that both sharks have pseudoosteodont teeth, but display differences in their vascularization that allow separation of these two groups. In this study we also were able to observe differences in the osteonal network of osteodont and pseudoosteodont teeth between different groups such as canal diameter and vascular density (fine reticulate networks in hexanchid sharks vs. very coarse networks in echinorhiniform sharks) and osteon arrangement (parallel, perpendicular dentinal osteons in molar teeth of *Heterodontus *vs. an irregular arrangement in lamniform sharks). Therefore, a phylogenetic signal cannot be disregarded here for the pseudoosteodont tooth histotype pending further studies about the tooth vascularization to resolve the presence or absence of such a signal.

The second most common tooth histotype, orthodonty, evolved three times independently in modern sharks: in the frilled shark *C. anguineus* (Hexanchiformes), in carpet sharks (Orectolobiformes), and in ground sharks (Carcharhiniformes). Orthodont teeth were also reported for other Chondrichthyes in †Xenacanthimorpha (Hampe & Ivanov, 2007; Ivanov, 2016) and †Hybodontiformes (Maisey et al. 2004). This indicates that the orthodont tooth histotype is a highly plastic feature that evolved several times independently in cartilaginous fishes and, therefore, makes phylogenetic interpretations of this histology pattern difficult.

In contrast to the previous two histotypes, the third histotype, osteodonty, is restricted to a single modern shark lineage, the lamniform sharks (Moyer et al. 2015; Schnetz et al. 2016; Jambura et al. 2019). Both species examined here, the extant longfin mako shark *Isurus paucus* and the fossil shark †*H. amonensis*, exhibited the osteodont tooth histotype. Of the 15 extant lamniform sharks, all except the basking shark *C. maximus* have osteodont teeth (Jambura et al. 2019). This unique pattern also has been reported for all examined extinct lamniform species so far, including †*Palaeocarcharias stromeri* from the Jurassic, whose systematic affiliation has long been discussed (de Beaumont, 1960; Duffin, 1988; Kriwet & Klug, 2004; Kriwet & Klug, 2015; Landemaine et al. 2018; Jambura et al. 2019). This tooth histology is known only from one other member of chondrichthyan fishes, the Palaeozoic shark †*Aztecodus harmsenae* (Hampe & Long, 1999). Lamniform sharks are specialized to a variety of different niches. The crocodile shark *Pseudocarcharias kamoharai*, a small deep‐sea shark, preys on small fishes and squids (Ebert & Mostrada, 2015), while lamnid sharks like the fast swimming mako sharks *Isurus* spp. and the great white shark *C. carcharias* also prey on bigger animals like swordfish and mammals (Cliff et al. 1989; Maia et al. 2006; Amorim et al. 2018). However, the filterfeeding megamouth shark *Megachasma pelagios* also belongs to this order (Taylor et al. 1983). Although they have very different lifestyles and prey preferences, they all share the same tooth histology, which strongly indicates that the osteodont tooth histology of lamniform sharks bears a phylogenetic signal rather than a functional signal.

### Functional signal

This study revealed a highly derived tooth mineralization pattern in the Port Jackson shark *H. portusjacksoni*, which exhibits two different histology patterns. Orthodentine is present in anterior teeth but restricted to the base of the tooth crown. The remainder of the tooth is composed of osteodentine. The enameloid covering the orthodentine is rather thin, while areas missing orthodentine are covered by an unusually massive enameloid. The histology of the posterior teeth differs from that of the anteriors. The orthodentine is reduced completely and only osteodentine is present, seemingly representing the osteodont histotype. The tooth mineralization pattern and the arrangement of dentinal osteons differs from the pattern found in lamniform sharks. Dentinal osteons are straight, parallel to each other and extending perpendicular to the coronal surface, leading to a columnar appearance. In contrast, osteodentine in lamniform sharks consists of more delicate osteons which are arranged irregularly and without clear patterns or directions.

Osteodentine of similar structure to that found in teeth of *H. portusjacksoni* were described for eagle rays [*Myliobatis* (Radinsky, 1961)], the cowtail stingray [*Pastinachus* (Adnet et al. 2018),)], and some hybodontiform shark‐like chondrichthyans [i.e. †*Acrodus*, †*Asteracanthus* and †*Tribodus* (Maisey et al. 2004)] and due to its columnar structure has been referred to as columnar osteodentine (Radinsky, 1961; Maisey et al. 2004). Because all these taxa are durophagous (consume hard‐shelled prey) and have similar tooth shapes (a somewhat rectangular outline with flat crown surfaces), an inherent link between their tooth composition, tooth morphology, and diet seems likely. This was also suggested in previous histological studies that pointed out differences of the enameloid of anterior (three layers) and posterior teeth (two layers) in heterodontids (Reif, 1973). The heterodont dentition of heterodontiform sharks makes them versatile feeders. The small cuspidate anterior teeth allow them to grasp soft‐bodied prey and detach fixed prey from the substrate, while the molariform posterior teeth allow them to crack hard‐bodied prey (Edmonds et al. 2001). Three‐dimensional static equilibrium analysis of the forces generated by the jaw musculature of the horn shark *Heterodontus francisci* also reflect these durophagous adaptations, with the maximum theoretical bite force at the anteriormost teeth being 128N, in contrast to 338N at the posterior molariform teeth (Huber et al. 2005). We therefore hypothesize that the columnar osteodentine in posterior teeth of heterodontiforms in combination with the tooth morphology are adaptations to a durophagous lifestyle. This is further supported by the ontogenetic dietary shift from juveniles to adults in heterodontiform sharks, which is accompanied by a change of the tooth morphology of the most distal tooth families (Reif, 1976; Powter et al. 2010). The dentition of the juveniles only consists of the small cuspidate teeth and they primarily prey on soft‐bodied invertebrates (although crustaceans were also found in their stomachs), while the posterior teeth of adults are broad and allow them to crush shelled prey (Reif, 1976; Powter et al. 2010).

The sawshark *P. nudipinnis* also has a highly modified tooth histology that does not reflect any of the three histotypes in sharks. No osteodentine could be identified in the root, and the crown has a hollow pulp cavity from which coarse dentinal tubes originated and are directed towards the apex. This pulp cavity‐dentinal tube system is surrounded by a thick layer of orthodentine. This histology is also known from many batoids and was referred to as orthodont due to its hollow pulp cavity (Herman et al. 1994). In the light of our results, we disagree with this interpretation, since this histology significantly differs from other orthodont sharks (e.g. Carcharhiniformes) in which osteodentine is present but restricted to the root. Therefore, we follow Peyer (1968) by referring to the tooth histology found in *Pristiophorus* and in rays (orthodentine in the crown and the root) as regular orthodont compared to the orthodont type in non‐pristiophoriform sharks. The presence of this very specialized tooth histology in both batoids and the sawshark also indicates a functional background rather than a phylogenetic signal.

### Conclusions

In this study we examined the tooth histology patterns in modern sharks of all nine extant orders plus members of the stem group †Synechodontiformes and investigated the evolutionary significance of the different tooth histologies. We were able to demonstrate that the pseudoosteodont tooth histology (osteodont core encapsulated by orthodentine) is the most widespread histotype and is present in all orders except for the sawsharks (Pristiophoriformes). The ancestral state analysis suggests the pseudoosteodont tooth histotype to be the plesiomorphic condition for all modern sharks (Fig. S1). From this basal state, two major evolutionary pathways in modern sharks were followed: the reduction of either osteodentine (orthodont histotype in the frilled shark *C. anguineus*, carcharhiniform, and some orectolobiform sharks) or orthodentine (osteodont histotype in lamniform sharks). The osteodont tooth histotype in lamniform sharks represents a unique histology pattern in lamniform sharks, which seemingly evolved independently from functional or ecological constraints. We therefore agree with previous studies that this histotype bears a strong phylogenetic signal.

There was no clear evidence for a functional signal of differently developed tooth histologies, except for pristiophoriform and heterodontiform sharks. *H. portusjacksoni* exhibits different tooth histologies in the anterior and posterior teeth, with the posterior teeth consisting of columnar osteodentine, which is most likely an adaptation to preying on hard‐shelled items. Teeth of the sawshark *P. nudipinnis* are very similar to batomorphs in appearance and histology, indicating that there is also a link between tooth microstructure, tooth morphology and lifestyle.

Although we were able to show that there is an evolutionary trajectory from pseudoosteodont to orthodont and osteodont teeth, we found that it was not possible to infer consistent phylogenetic or functional signals from the different tooth histotypes for all groups of sharks. The present study suggests that this information could be found in the plethora of different mineralization patterns that are described here, but the terms orthodont, osteodont and pseudoosteodont are overused descriptors for a broad range of histology patterns and are best thought of being extremes of a histological continuum. Further studies incorporating additional micromorphological data will be needed to fully resolve the relationships between tooth histology, morphology and its possible implications for the function of the tooth.

## Conflict of interest

None declared.

## Supporting information


**Fig. S1.** Ancestral state reconstruction for the tooth histology patterns in modern sharks.

**Table S1.** List of examined species and additional information.

**Table S2.** Applied CT devices and settings for the examined material.

## Data Availability

The raw data that support the findings of this study are available from the corresponding author or the co‐author J.K. upon reasonable request.
