# Supplementary figures and images for: Evolutionary trajectories of tooth histology patterns in modern sharks (Chondrichthyes, Elasmobranchii)
Source: J Anat. 2019 Dec 22;236(5):753–71. doi: 10.1111/joa.13145 (PMC7163786; doi:10.1111/joa.13145)

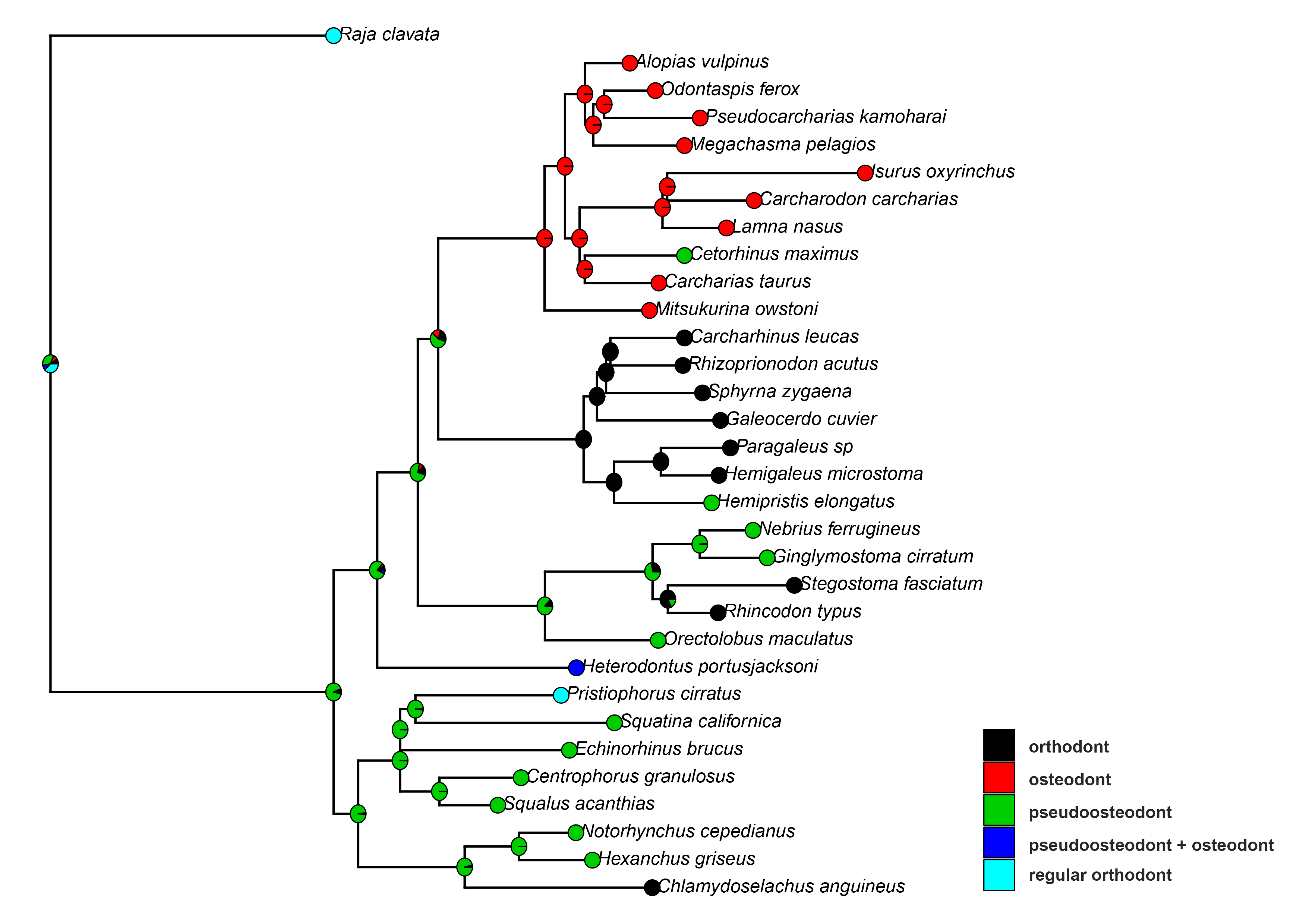

Supplement: Supplementary file 1 — Fig. S1. Ancestral state reconstruction for the tooth histology patterns in modern sharks. [file JOA-236-753-s001.tif]
