# Supplementary material for: Evolutionary trajectories of tooth histology patterns in modern sharks (Chondrichthyes, Elasmobranchii)
Source: J Anat. 2019 Dec 22;236(5):753–71. doi: 10.1111/joa.13145 (PMC7163786; doi:10.1111/joa.13145)
Supplement: Supplementary file 2 — Table S1. List of examined species and additional information. [file JOA-236-753-s002.docx]

| Species | Inventory-No | Material | Age | Locality | Collection |
| --- | --- | --- | --- | --- | --- |
| *Centrophorus granulosus* | EMRG-Chond-T-62 | tooth | recent | Phillipines, Pacific | University of VIenna |
| *Chlamydoselachus anguineus* | EMRG-Chond-T-66 | tooth | recent | South-Atlantic | University of VIenna |
| *Echinorhinus brucus* | EMRG-Chond-J-19 | jaw | recent | - | University of VIenna |
| *Echinorhinus cookei* | EMRG-Chond-T-64 | tooth | recent | New Zealand | University of VIenna |
| †*Galeocerdo mayumbensis* | 7-713 | tooth | Miocene | Peace River Formation, Bone Valley, Florida | Haimuseum und Sammlung R. Kindlimann |
| †*Haimirichia amonensis* | 7-09 | tooth | Cenomanian, Cretaceous | Woodbine Formation, Amon Carter Airfield, Texas | Haimuseum und Sammlung R. Kindlimann |
| *Heterodontus portusjacksoni* | EMRG-Chond-J-20 | tooth | recent | - | University of VIenna |
| *Heterodontus portusjacksoni* | EMRG-Chond-J-20 | jaw | recent | - | University of VIenna |
| *Heterodontus portusjacksoni* | EMRG-Chond-T65 | tooth | recent | - | University of VIenna |
| †*Hexanchus microdon* | EMRG-Chond-T-38 | tooth | Ypresian, Eocene | Ouled Abdoun, Morocco | University of VIenna |
| *Isurus paucus* | 7-715/RZ | tooth | recent | Pacific,Phillipines | Haimuseum und Sammlung R. Kindlimann |
| †*Nebrius blanckenhorni* | 1978/1966/0024a | tooth | Ypresian, Eocene | Khouribga, Morocco | Natural History Museum Vienna |
| †*Notorynchus kempi* | 2006z0274/0001 | tooth | Bartonian, Eocene | Usak, Mangyshlak, Kasachstan | Natural History Museum Vienna |
| *Orectolobus maculatus* | EMRG-Chond-T-67 | tooth | recent | Bunbury Western Australia | University of VIenna |
| †*Paraorthacodus* sp. | SMNS-87088 | tooth | upper Albian, Cretaceous | Stary Oskol, Russia | State Museum of Natural History Stuttgart |
| †*Physogaleus* sp. | 7-716 | tooth | Ypresian, Eocene | Nanjemoy Formation, Fredericksburg, Virginia | Haimuseum und Sammlung R. Kindlimann |
| *Pristiophorus nudipinnis* | EMRG-Chond-T-61 | tooth | recent | Australia | University of VIenna |
| *Rhincodon typus* | 7-714/RZ | tooth | recent | Indo-Pacific, Indonesia | Haimuseum und Sammlung R. Kindlimann |
| †*Rhomphaiodon minor* | EMRG-Chond-T-40 | tooth | Rhaetian, Late Triassic | Bebenhausen, Tübingen, Germany | University of VIenna |
| †*Rhomphaiodon minor* | EMRG-Chond-T-41 | tooth | Rhaetian, Late Triassic | Bebenhausen, Tübingen, Germany | University of VIenna |
| *Squalus acanthias* | EMRG-Chond-T-63 | tooth | recent | - | University of VIenna |
| †*Squatina angeloides* | EMRG-Chond-T-68 | tooth | Oligocene | Neumühle bei Alzey, Rheinlandpfalz, Germany | University of VIenna |
| †*Squatina prima* | EMRG-Chond-T-69 | tooth | mid Eocene | Tusbair, Mangyshlak, Kazachstan | University of VIenna |
| *Squatina squatina* | EMRG-Chond-J-17 | tooth | recent | Atlantic | University of VIenna |
| †*Squatina subserrata* | 2005z0305/0087a | tooth | Ottnangium, Miocene | Passau, Germany | Natural History Museum Vienna |
| †*Synechodus* sp. | SMNS-87099 | tooth | Upper Albian, Cretaceous | Stary Oskol, Russia | State Museum of Natural History Stuttgart |
