# Supplementary material for: Evolutionary trajectories of tooth histology patterns in modern sharks (Chondrichthyes, Elasmobranchii)
Source: J Anat. 2019 Dec 22;236(5):753–71. doi: 10.1111/joa.13145 (PMC7163786; doi:10.1111/joa.13145)
Supplement: Supplementary file 3 — Table S2. Applied CT devices and settings for the examined material. [file JOA-236-753-s003.docx]

| Species | Inventory No. | Material | CT-device | Voxel size [µm] | Source-Voltage [kVp] | Source current [µA] | Beam Filter | Exposure time [ms] | Frame averaging |
| --- | --- | --- | --- | --- | --- | --- | --- | --- | --- |
| *Centrophorus granulosus* | EMRG-Chond-T-62 | tooth | Xradia MicroXCT | 4.01 | 80 | 62 | 0.5mm Al | 7500 | - |
| *Chlamydoselachus anguineus* | EMRG-Chond-T-66 | tooth | SkyScan1173 | 7.15 | 80 | 100 | No Filter | 650 | 3 |
| *Echinorhinus brucus* | EMRG-Chond-J-19 | jaw | SkyScan1173 | 27.90 | 50 | 160 | User Filter | 1100 | 3 |
| *Echinorhinus cookei* | EMRG-Chond-T-64 | tooth | SkyScan1173 | 7.15 | 80 | 100 | No Filter | 650 | 3 |
| †*Galeocerdo mayumbensis* | 7-713 | tooth | SkyScan1173 | 17.88 | 100 | 80 | Al 1.0mm | 750 | 4 |
| †*Haimirichia amonensis* | 7-09 | tooth | SkyScan1173 | 12.16 | 80 | 100 | No Filter | 650 | 4 |
| *Heterodontus portusjacksoni* | EMRG-Chond-J-20 | tooth | Xradia MicroXCT | 2.10 | 60 | 82 | LE#1 0.25mm glass | 18000 | - |
| *Heterodontus portusjacksoni* | EMRG-Chond-J-20 | jaw | SkyScan1173 | 17.17 | 80 | 100 | No Filter | 650 | 3 |
| *Heterodontus portusjacksoni* | EMRG-Chond-T65 | tooth | SkyScan1173 | 8.94 | 80 | 100 | No Filter | 650 | 3 |
| †*Hexanchus microdon* | EMRG-Chond-T-38 | tooth | SkyScan1173 | 7.15 | 80 | 100 | No Filter | 650 | 3 |
| *Isurus paucus* | 7-715/RZ | tooth | SkyScan1173 | 23.96 | 80 | 100 | No Filter | 650 | 3 |
| †*Nebrius blanckenhorni* | 1978/1966/0024a | tooth | SkyScan1173 | 7.15 | 80 | 100 | No Filter | 650 | 3 |
| †*Notorynchus kempi* | 2006z0274/0001 | tooth | SkyScan1173 | 17.17 | 80 | 100 | No Filter | 650 | 3 |
| *Orectolobus maculatus* | EMRG-Chond-T-67 | tooth | SkyScan1173 | 10.02 | 80 | 100 | No Filter | 650 | 2 |
| †*Paraorthacodus* sp. | SMNS-87088 | tooth | SkyScan1173 | 7.13 | 80 | 100 | No Filter | 650 | 5 |
| †*Physogaleus* sp. | 7-716 | tooth | SkyScan1173 | 12.16 | 100 | 80 | Al 1.0mm | 750 | 4 |
| *Pristiophorus nudipinnis* | EMRG-Chond-T-61 | tooth | Xradia MicroXCT | 4.01 | 80 | 62 | LE#1 0.25mm glass | 5000 | - |
| *Rhincodon typus* | 7-714/RZ | tooth | SkyScan1173 | 11.09 | 80 | 100 | No Filter | 650 | 3 |
| †*Rhomphaiodon minor* | EMRG-Chond-T-40 | tooth | SkyScan1173 | 7.15 | 80 | 100 | No Filter | 650 | 3 |
| †*Rhomphaiodon minor* | EMRG-Chond-T-41 | tooth | SkyScan1173 | 7.15 | 80 | 100 | No Filter | 650 | 3 |
| *Squalus acanthias* | EMRG-Chond-T-63 | tooth | Xradia MicroXCT | 4.01 | 80 | 62 | LE#1 0.25mm glass | 5000 | - |
| †*Squatina angeloides* | EMRG-Chond-T-68 | tooth | SkyScan1173 | 7.51 | 80 | 100 | No Filter | 650 | 3 |
| †*Squatina prima* | EMRG-Chond-T-69 | tooth | SkyScan1173 | 7.51 | 80 | 100 | No Filter | 650 | 3 |
| *Squatina squatina* | EMRG-Chond-J-17 | tooth | VISCOM X8060 NDT | 25.00 | - | - | - | - | - |
| †*Squatina subserrata* | 2005z0305/0087a | tooth | SkyScan1173 | 7.15 | 80 | 100 | No Filter | 650 | 3 |
| †*Synechodus* sp. | SMNS-87099 | tooth | SkyScan1173 | 7.13 | 80 | 100 | No Filter | 650 | 5 |
